# Demographic Imbalances Resulting From the Bring-Your-Own-Device Study Design

**DOI:** 10.2196/29510

**Published:** 2022-04-08

**Authors:** Peter Jaeho Cho, Jaehan Yi, Ethan Ho, Md Mobashir Hasan Shandhi, Yen Dinh, Aneesh Patil, Leatrice Martin, Geetika Singh, Brinnae Bent, Geoffrey Ginsburg, Matthew Smuck, Christopher Woods, Ryan Shaw, Jessilyn Dunn

**Affiliations:** 1 Department of Biomedical Engineering Duke University Durham, NC United States; 2 Clinical and Translational Science Institute Duke University Durham, NC United States; 3 All of Us Research Program National Institutes of Health Bethesda, MD United States; 4 Department of Physical Medicine and Rehabilitation The Spine Program University of Michigan Ann Arbor, MI United States; 5 Division of Infectious Diseases Duke University Medical Center Durham, NC United States; 6 School of Nursing Duke University Durham, NC United States; 7 Department of Biostatistics and Bioinformatics Duke University Durham, NC United States

**Keywords:** bring your own device, wearable device, mHealth

## Abstract

Digital health technologies, such as smartphones and wearable devices, promise to revolutionize disease prevention, detection, and treatment. Recently, there has been a surge of digital health studies where data are collected through a bring-your-own-device (BYOD) approach, in which participants who already own a specific technology may voluntarily sign up for the study and provide their digital health data. BYOD study design accelerates the collection of data from a larger number of participants than cohort design; this is possible because researchers are not limited in the study population size based on the number of devices afforded by their budget or the number of people familiar with the technology. However, the BYOD study design may not support the collection of data from a representative random sample of the target population where digital health technologies are intended to be deployed. This may result in biased study results and biased downstream technology development, as has occurred in other fields. In this viewpoint paper, we describe demographic imbalances discovered in existing BYOD studies, including our own, and we propose the Demographic Improvement Guideline to address these imbalances.

## Introduction

Digital health tools, including mobile health (mHealth) and wearable devices, can provide researchers with high-frequency data that are more representative of a person’s health state during their daily life than what can be collected in a clinical setting [1,2]. The enormous benefits of acquiring data outside of the clinic have led researchers to adopt new study designs to incorporate digital health data collection tools. Digital biomarkers constitute a type of biomarker that is developed from digitally collected data, such as wearable devices and smartphones, to evaluate functions and processes in the body and that can typically be recorded outside of a lab setting to provide continuous and more holistic information [3]. In particular, the bring-your-own-device (BYOD) study design has become increasingly popular because it gives researchers the ability to collect large-scale data at a low cost from participants who already own personal electronic devices, such as smartphones and wearable devices. During the COVID-19 pandemic, there has been growing interest in using digital health data to track illness, either for COVID-19 detection or to support telemedicine [4-6]. To process all this data, artificial intelligence algorithms, specifically machine learning algorithms, are being developed to detect health conditions by learning from previously collected data.

Machine learning algorithms rely on data to train models; they are susceptible to biases that result in poor predictions for segments of the population if the training data are not representative of the population where the model is intended to be used [7]. Therefore, one key aspect of machine learning is the data collection process, whereby researchers identify the target population and select a representative, random sample of the population from which to collect unbiased data [8]. BYOD studies are particularly susceptible to bias in the data collection process because the recruitment pool is limited to people who already own a device, and this population is generally not the only one where the tools are ultimately intended to be used in practice. In BYOD studies, a nonrepresentative study population excludes key socioeconomic and physiologic circumstances that can covary with race, ethnicity, or both. For example, disease prevalence and pathophysiology often vary by race and ethnicity (eg, COVID-19 infection and mortality rates [9-11], manifestation of metabolic disease [12-14], cardiovascular disease [15,16], and sleep irregularities [17,18]), which can result in differences in how the newly developed technologies will perform. As a specific example, optical sensors to measure blood oxygen saturation may fail in people with more melanin and for people with the sickle cell trait [19-21], both characteristics common in Black populations, for two completely separate reasons. To address such problems, representative digital health data are needed for building generalizable machine learning models that are as accurate under deployment as they are in the initial testing phase. Like other fields that have discovered that bias in data used to train models has led to biased models, we fear that digital health will face similar challenges if the bias inherent in BYOD studies is left unaddressed [22,23]. In this viewpoint paper, we seek to raise awareness of demographic imbalances in several BYOD studies and propose a guideline based on our own BYOD case study to directly improve the demographic balance of BYOD digital health studies.

## Demographic Imbalances in Existing BYOD Studies

BYOD is a term used to describe studies in which participants contribute data from self-owned personal electronic devices. We collected a sample of BYOD studies using multiple search terms on PubMed and Web of Science, including “bring your own device,” “(consumer) wearable device study,” “remote mobile study,” and “mHealth,” and performed manual review and filtering, which generated 15 relevant studies. Although we note that this is not a systematic approach to compiling all published BYOD studies, and we acknowledge the potential to overlook studies that have successfully recruited a representative study population, here we demonstrate that many existing BYOD studies have gender and race imbalances when compared to the broader US demographic profile. Of the 15 studies identified, 4 (27%) did not report any demographics on ethnicity or race, and none of the remaining 11 (73%) studies achieved participant demographic proportions representative of the overall US demographic profile (Multimedia Appendix 1).

**Figure 1:**
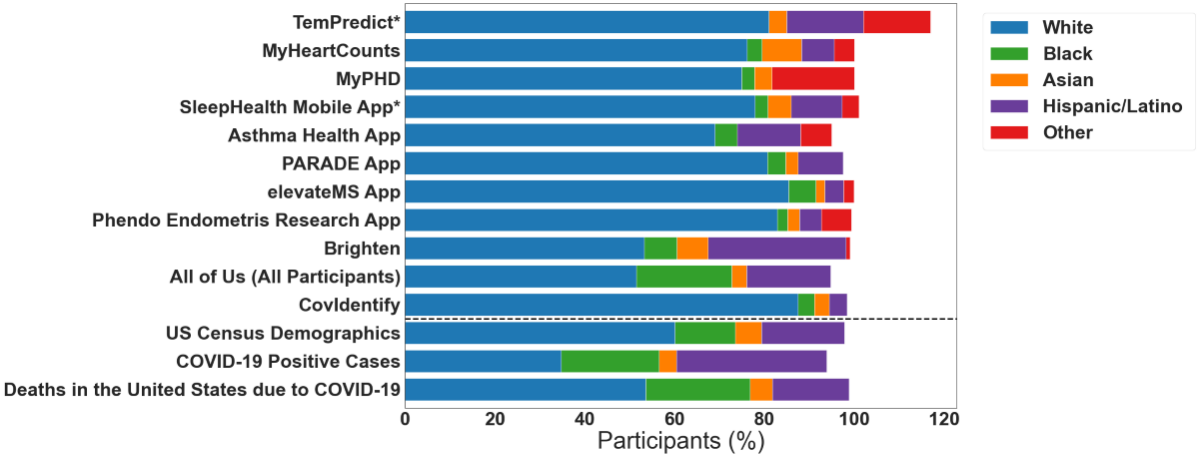
Comparing demographic distributions from various bring-your-own-device (BYOD) studies (listed on the y-axis, above the dotted line), the national census, and the National Vital Statistics System. Studies with an asterisk did not separate ethnicity and race and, therefore, have percentages that sum up to be greater than 100. Other studies did not report a breakdown for all the ethnicity and race groups and, therefore, resulted in an aggregated percentage less than 100.

One of the most pre-eminent BYOD studies was a substudy of the All of Us research initiative [24] by the US National Institutes of Health (NIH). In that substudy, wearable device data were collected from participants who owned Fitbit devices between 2008 and 2019 and who consented to share their data. More than 80% of participants in the overarching All of Us study were from historically underrepresented groups in biomedical research. As a strong juxtaposition, 70% of the participants in the All of Us BYOD substudy identified as White non-Hispanic, while only 4% and 3% identified as Black and Asian, respectively (Figure 1 and Multimedia Appendix 2). The ethnicity distribution in the All of Us study tells a similar story, with over 90% of the participants identifying as non-Hispanic or non-Latino, and only 6% identifying as Hispanic or Latino. Even in this diverse, large-scale study that had specifically targeted recruitment toward underrepresented groups, equitable demographic representation was limited by the BYOD study design.

Similar to other BYOD studies, we discovered demographic imbalances in our own CovIdentify study (Multimedia Appendices 3 and 4). The unique circumstances of the evolving COVID-19 pandemic led to a rapid launch of our study, where we aimed to develop an intelligent testing strategy using digital biomarkers extracted from personally owned commercial wearable devices under resource-constrained conditions (limited testing, rural areas, etc). However, after our rapid study launch, we found that the communities most vulnerable to COVID-19 [9,25] were not well-represented in our study.

To mitigate this demographic imbalance and to ensure the inclusion of participants from underserved communities, we developed the Demographic Improvement Guideline and correspondingly altered our recruitment process. Although we developed this guideline retrospectively after the discovery of demographic imbalances in our study, we are calling for future research to take proactive measures during the BYOD study design as well as responsive measures during participant recruitment and retention efforts. Many BYOD studies acknowledge demographic imbalance as a limitation, and we believe a concerted effort is needed to enact change to reduce bias in digital health data used in research.

## Demographic Improvement Guideline

### Overview

The goal of BYOD studies is to develop new device-based technologies and interventions to improve the health or well-being of populations. In order for these interventions to be fit for purpose, the research and development should include the populations where the technologies will ultimately be used, or their exclusion should be well-justified and should not introduce bias or harm. There may be cases where demographic imbalances (ie, sampling bias) would not be problematic in a BYOD study, for example, if both of the following conditions apply: (1) the manifestation of the disease as measured by the wearable device does not differ across race, ethnicity, or age and (2) the technology works the same for all people across the entire population. In such cases, the researcher would not need to focus efforts and resources on obtaining a representative demographic distribution in the study population.

However, it is known that (1) most diseases do not manifest in the same way across different demographics and (2) smart devices do not work equally well or are not equally accessible across all demographics. As such, we cannot conclude that a biased sampling strategy is generally an appropriate choice. There will certainly be exceptions where the study population is appropriately focused on a certain group (eg, only including females in pregnancy-related studies), which can be achieved by applying inclusion and exclusion criteria for study participation. In this case, before a biased sampling strategy is chosen, the researcher is obligated to prove the null hypothesis for differences in disease manifestation and device function between the biased sample and the population where the technology is intended to be used. We purport that this step is often an even larger barrier than designing an equitably sampled study population. Next, we describe two concrete examples of times when equitable sampling was not prioritized and resulted in incorrect study conclusions.

The first condition for considering sampling bias is when domain knowledge from a field gives a priori indication that disease prevalence and pathophysiology may vary by race, ethnicity, or both, for example, COVID-19 infection and mortality rates, metabolic disease, cardiovascular disease, and sleep disturbances, among others [9-18]. Coronary artery disease is a historical example of the unintended and harmful effects that biased study populations can have on study conclusions: most early coronary artery disease studies consisted of homogenous male populations and, as a result, differences in symptoms for men and women were not discovered until follow-up studies included female populations [26,27]. Often there is insufficient a priori knowledge of differences in disease manifestation across age, gender, race, ethnicity, etc. As a result, the effect of biased sampling on study conclusions is frequently unknown. Furthermore, data quality and sampling often vary across demographics, particularly in mobile and wearable device studies, which further complicates determining the most appropriate sampling method. For example, use of wearable devices, ranging from commercial wearable devices to more sophisticated health monitoring devices, is lower among adults over 50 years of age compared to adults aged 18 to 34 years. Young, healthy, and more educated individuals are more likely to own wearable devices [28].

The second condition for considering sampling bias is when the technology used for data collection has not been validated systematically (ie, it has either not reported demographic distributions of the test population or has uncertainty in its measurements). For example, a study published in 2020 in the New England Journal of Medicine compared values of blood oxygen saturation in occult hypoxia measured via pulse oximetry with arterial oxygen saturation in arterial blood gas, the gold standard, to determine the validity of the pulse oximetry measurements [19]. The study obtained nearly 50,000 pairs of measurements from more than 8000 White patients and 1000 Black patients. The frequency of occult hypoxia detection via pulse oximetry was three times lower for Black patients than White patients. Given the prevalent use of pulse oximetry in medical decision-making, the implication of sensors reporting varying results based on an individual’s skin tone is concerning. The study points to the need for manufacturers of optical heart rate and blood oxygen saturation sensors to disclose the demographic distribution of the populations that were used to test the sensors. Since the primary sensor on most of the wrist-worn commercial wearable devices (eg, Apple Watch, Fitbit, and Garmin) relies on a similar optical sensor, ensuring that the measurements are accurate for anyone who wears the device is crucial. Because the performance of technology across demographic characteristics is not systematically evaluated and published for most commercial wearable devices, the technology and models derived from them may fail to generalize across demographic characteristics [20,29].

This guideline is relevant to studies in which sampling bias resulting in demographic imbalance could challenge the validity and generalizability of a BYOD study’s conclusions. The method can be implemented iteratively in the study design and execution process, and includes the following steps:

1. Identify one or more populations at risk of being omitted from the study for whom the technology may ultimately be used and determine if BYOD study design is appropriate for the research question.
2. If the BYOD study design is insufficient for addressing issues associated with demographic imbalance, modify the study design using internal and external resources to improve dissemination of information and improve engagement with the target populations.
3. Launch the study and monitor study demographics in real time to adjust downstream efforts accordingly.

### Identify Populations of Interest

To identify the populations that are at risk of being omitted, a literature review can reveal a baseline expectation of demographic distributions from prior studies using similar devices and advertisement strategies. To support this, it is necessary for publication venues and funding agencies to require detailed demographic reporting of BYOD studies. In addition to the proactive measures mentioned above, researchers should conduct early and ongoing systematic analyses of their study demographics and iteratively adapt their strategies accordingly.

### Modify Study Design

Capabilities to disseminate information, provide physical resources, and improve engagement with the target population can be assessed and developed internally and externally. Internal resources may involve organizations within the research institution that have experience recruiting underrepresented populations or have ties with the underrepresented groups. Another internal approach may be choosing the devices to be included in the study, considering whether these devices have widespread use in the underrepresented groups, and augmenting and using capabilities of available devices (eg, using sensors of more widely available smartphones to capture physiology and activity characteristics instead of consumer wearables) [30]. External resources may include community groups that are representative of the target population, government and nongovernmental organizations that work with the underrepresented communities, or donors who can donate devices that can be distributed to the target population. Other external resources may include clinician referral, which can help establish trust with the target population and improve retention of study participants [31].

For national or international studies, researchers can partner with nationwide organizations, launch social media advertising campaigns, or both [32-34]. For regional studies, researchers can connect with institutional and local resources to learn about and connect with community groups. Researchers may recruit a liaison to aid in establishing partnerships with external organizations and establish a community advisory board to interact directly with community groups to contribute to the study design and advocate for participation in the study.

One existing challenge is a dearth of funding to support equitable digital health study design, including the purchase of personal electronic devices, such as wearables and smartphones. Funders should be aware of this challenge and develop funding opportunities to support equitable digital health research. Given that acquiring funding is a well-known challenge, there are methods by which researchers can alter study design to reduce costs. This can include altering the study to follow a subset of the study population based on specific cases and controls. One method is the implementation of a randomized withdrawal design where participants who are inactive at the beginning of the study are excluded so that participants who are active are presented in the actual randomized study [35]. This could limit the number of participants that researchers need to follow up on and provide a more robust data set, but this may also skew demographics. Another challenge is to ensure that the distributed devices are used as intended, given that they require a certain level of digital literacy and need to be consistently worn and charged over time. As such, participant compensation amount and timing as well as wear tracking should be included as considerations in the study design and should be supported by funding agencies [36].

### Launch Study and Reassess Study Population

After study modifications, the study is ready for deployment. Depending on the funding received and study design changes, the resulting study may be a hybrid study where devices are provided for the target population. During this launch phase, researchers should regularly assess their demographic distributions to determine whether their strategy is effective. If the strategy is not effective, researchers can restrategize with their internal and external community partners and identify aspects of the study that may be deterring participant engagement or recruitment. Finally, study teams should continue to engage with the study population after the end of the study to convey findings and future opportunities for participation in research.

## A Case Study on Practically Implementing the Demographic Improvement Guideline

### Overview

The following subsections provide details about the responsive implementation of the Demographic Improvement Guideline after the launch of the CovIdentify study (Institutional Review Board No. 2020-0412). The study aimed to develop machine learning algorithms to detect COVID-19 and influenza from wearable device data, with a long-term vision of developing an intelligent diagnostic testing strategy using digital biomarkers extracted from personally owned commercial wearable devices under resource-constrained conditions (limited testing, rural areas, etc). In April 2020, CovIdentify began enrolling participants. Following informed consent, participants contributed their wearable device data (eg, Fitbit, Garmin, and Apple Watch) and reported daily symptoms for 12 months via a downloadable app, email, or text message.

### Identify

**Figure 2:**
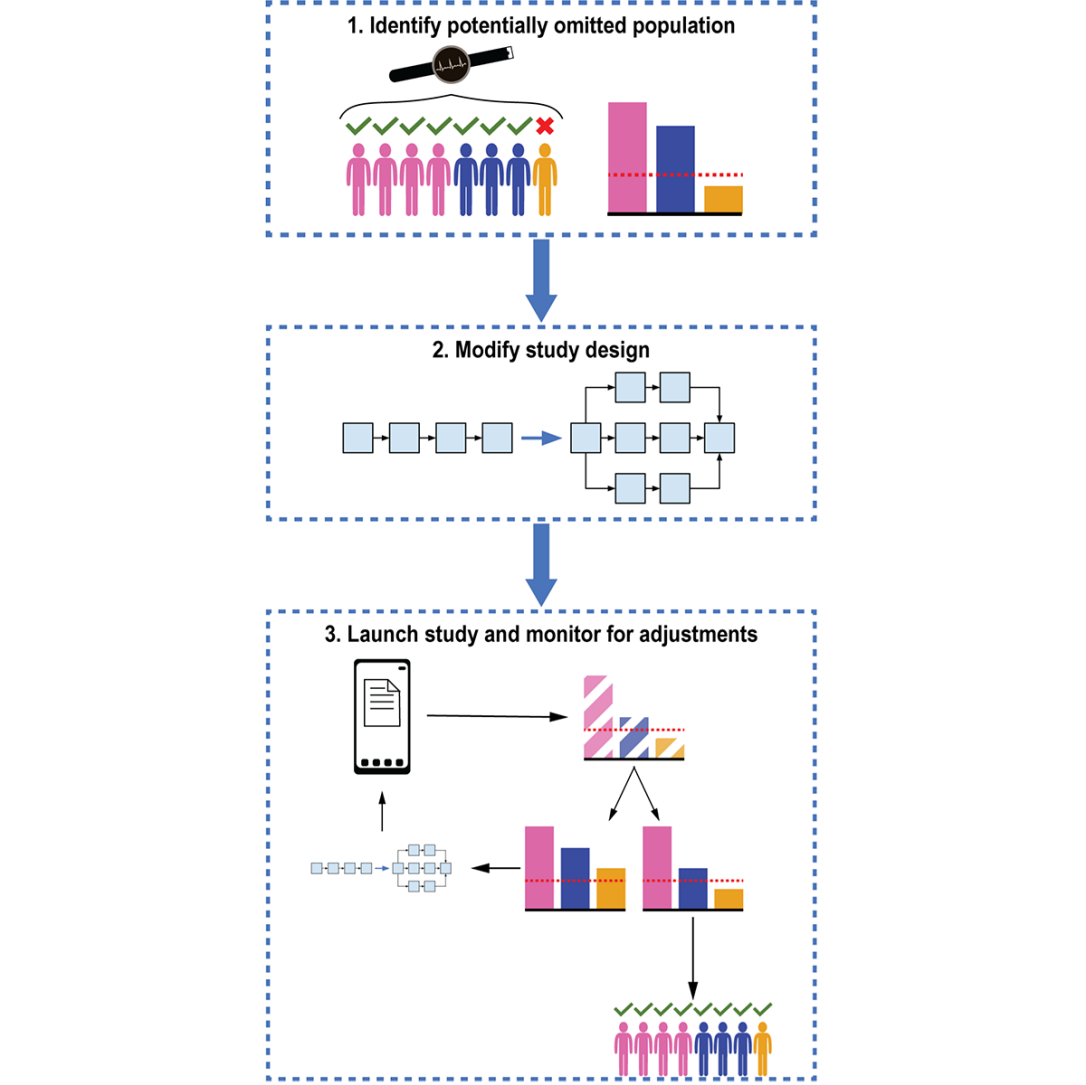
Visualization of the Demographic Improvement Guideline. Step 1. Identify the populations at risk of being omitted from the study and for whom the technology may ultimately be used. Step 2. Modify study design based on internal and external resources to disseminate information and improve engagement with the target populations. Step 3. Launch the study, monitor study demographics in real time, and adjust downstream efforts accordingly. Researchers should reassess their study population demographics to ensure that target distributions are achieved and restrategize accordingly. The red dashed lines in the bar charts are visual representations of the acceptable threshold for each subgroup population.

Following the rapid launch, exploratory data analysis revealed major differences between CovIdentify demographics and the demographics of COVID-19–positive cases and deaths in the United States [30], as well as overall US demographics based on the 2020 US Census. The communities hardest hit by the COVID-19 pandemic, including Black and African American as well as Hispanic and Latinx communities, also had the lowest representation (Figure 2) [9,37,38].

### Modify

To address this imbalance, we designed the Demographic Improvement Guideline mitigation strategy in partnership with Duke University’s Clinical and Translational Science Institute (CTSI), an NIH-funded center that connects researchers with local community partners and improves the reach and efficacy of research [39]. We piloted this method in the Durham, North Carolina community from June through October of 2020. The CTSI facilitated connections between our research team and local community and faith leaders, including groups working to address COVID-19–related health disparities affecting the Latinx and African American communities. This engagement enabled our team to learn directly from community members, researchers, and health care professionals in order to improve our advertisement and recruitment strategies to (1) increase awareness about the study and (2) distribute wearable devices that were donated to our study and purchased through research funding. To expand awareness of the study, our team gave presentations to community groups regarding the advantages of continuous health monitoring, the uses of participant information in this study, and imbalances in our current study population that would make it difficult for our team to develop generalizable study findings. We also recruited a liaison to the Latinx community, translated our website to four additional languages, ran multilingual social media advertisements featuring diverse images and videos, and posted messages about the study on various social media platforms.

### Launch Study and Reassess Study Population

**Figure 3:**
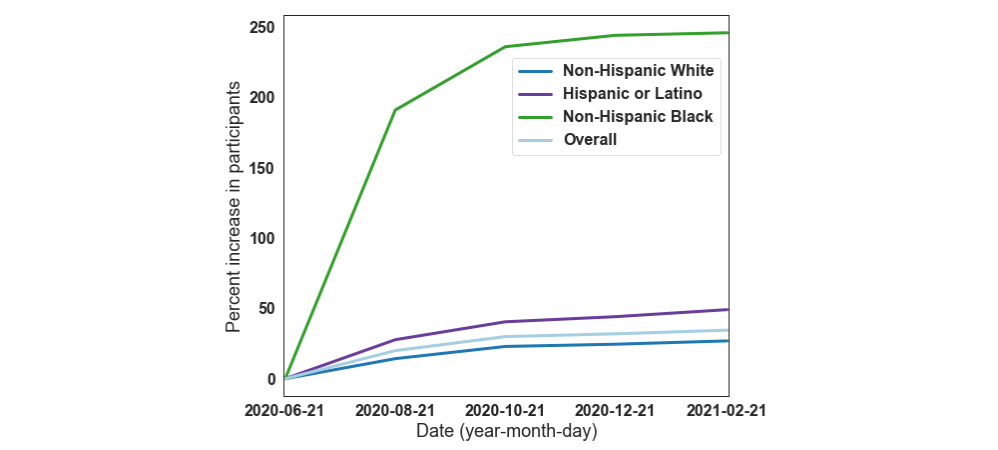
Percent increase in CovIdentify study population participants compared to June 21, 2020.

To support the purchase of devices and social media advertising campaigns, we applied for nearly 30 funding opportunities from government, nonprofit, and industry sources. We were awarded a Duke Bass Connections Fellowship, a North Carolina Biotechnology Institute grant, and a Duke MEDx/CTSI award that enabled us to purchase 65 wearable devices for distribution. We also received a donation of 300 additional devices from a previously completed study. We attended 12 community events, including food and medication distribution events for low-income members of the Durham community, and distributed 250 free wearable devices in a socially distant manner to ensure safety during the COVID-19 pandemic. It should be noted that this was a hybrid BYOD design because participants were still required to own a smartphone to connect their wearable device to the study. We also worked with wearables companies to set up reduced device pricing with a direct link from our study’s main webpage to improve accessibility. Together, these efforts resulted in a 250% increase in the representation of Black and African American participants and a 49% increase in the Latinx and Hispanic population within 4 months of the implementation of the guideline (Figure 3).

## Discussion

In this viewpoint paper, we discussed the need for digital health studies to sample from populations representative of the target population to ensure equitable performance of predictive or machine learning models. We explored demographic imbalances in BYOD studies and proposed the Demographic Improvement Guideline to address these imbalances with an implementation example from the CovIdentify study. We believe that future efforts and funding in this space can further improve equitable digital health study design and data collection. Further, we recommend that researchers carefully consider the financial incentives and personal motivations provided to participants by the study to identify driving factors for participation and engagement.

By developing the Demographic Improvement Guideline, we aim to facilitate improvement of future BYOD study design through fostering relationships and trust with local community groups. These methods are not limited to BYOD studies only. We can translate these methods to non-BYOD studies as well. In addition, we believe that implementing existing community-based engagement methods, such as training recruiters and providing face-to-face screening, can improve both recruitment methods and adherence to studies [40-43]. We also emphasize the need for increased funding opportunities in this area to enable the development of equitable algorithms and models that are representative of all individuals.

One limitation of the Demographic Improvement Guideline was the condition under which it was developed. While the guideline is a proactive recommendation, the implementation within our case study was retrospective to our findings. Therefore, shifts in our study design, such as donating commercial wearable devices to underrepresented groups, have resulted in a non-BYOD (ie, hybrid) study. Furthermore, due to the evolving nature of the pandemic and the resulting rapid launch of the CovIdentify study, factors mentioned in the guideline may not be applicable to all study designs. Here, we intend to present the guideline as one unique potential solution of the many possible solutions to address demographic imbalances in BYOD studies. This experience underscores the importance of addressing potential demographic inequities prior to a BYOD study launch.

## Appendix

Multimedia Appendix 1 Race and ethnicity breakdown from bring-your-own-device (BYOD) studies.

Multimedia Appendix 2 Sex, race, and ethnicity reported by various bring-your-own-device (BYOD) studies, the national census, and the National Vital Statistics System.

Multimedia Appendix 3 Wearable device ownership by race or ethnicity for CovIdentify case study.

Multimedia Appendix 4 Wearable device ownership of iPhone (iOS) users by race or ethnicity for CovIdentify case study.

## Abbreviations

BYOD: bring your own device
CTSI: Clinical and Translational Science Institute
mHealth: mobile health
NIH: National Institutes of Health
