# Supplementary material for: Demographic Imbalances Resulting From the Bring-Your-Own-Device Study Design
Source: JMIR Mhealth Uhealth. 2022 Apr 8;10(4):e29510. doi: 10.2196/29510 (PMC9034431; doi:10.2196/29510)
Supplement: Multimedia Appendix 1 [file mhealth_v10i4e29510_app1.docx]

**Multimedia Appendix 1**

**Table S1.** Race and Ethnicity Breakdown from BYOD studies

| Paper Title | Wearable Device Requirement for Participation | Open to/Targeted Communities | Demographic information collected | Sample Size | Age Distribution | Gender Distribution | Ethnicity/Race Distribution |
| --- | --- | --- | --- | --- | --- | --- | --- |
| Comparison of On-Site Versus Remote Mobile Device Support in the Framingham Heart Study Using the Health eHeart Study for Digital Follow-up: Randomized Pilot Study Set Within an Observational Study Design [1] | Fitbit device, iHealth blood pressure cuff, iHealth scale, AliveCor | “Co Enrolled participants from the Framingham Heart Study (FHS) into the FHS–Health eHeart (HeH) pilot study, a digital cohort with infrastructure for collecting mHealth data. FHS participants who had an email address and smartphone were randomized” | Age, Gender, Education, Body Mass Index, Physical Activity Index  “Oversampled older adults (age ≥65 years), with a target of enrolling 20% of our sample as older adults.” | n = 194  Group 1 *(randomized to on-site)*  n = 101  Group 2  *(randomized to remote)*  n = 93 | Age (years), mean (SD)  Group 1: 55 (11)  Group 2: 53 (10) | Women, n (%)  Group 1: 60 (59)  Group 2: 57 (61)  Men, n (%)  Group 1: 41 (41)  Group 2: 36 (39) | Exact numbers were unreported; however, participants from the Framingham Heart Study consisted mostly of white individuals from New England |
| Feasibility of continuous fever monitoring using wearable devices [2] | “Oura smart ring that pairs with Oura app on a user’s smartphone” | “Launched TemPredict study in March of 2020 to capture continuous physiological data… report findings from the first 50 subjects who reported COVID-19 infections”. | Residence, Vocation, Gender, Age, Household size, Education, Race | n = 50 | Age (years), mean (SD)  43.7 (11.0) | Women, n (%)  17 (34)  Men, n (%)  33 (66) | # of participants reporting race n = 48  White, n (%)  39, (81)  Black, n (%)  0 (%)  Asian, n (%)  2 (4)  Other, n (%)  4 (6)  Latino, n (%)  8 (17)  Non-Latino, n (%)  40 (83) |
| Four-Year Trends in Sleep Duration and Quality: A Longitudinal Study Using Data from a Commercially Available Sleep Tracker [3] | Sleep Cycle app on a smartphone | “Eligibility criteria for this study included living in a major urban center (New York City) and age 13 years or older.” | Age, sex, sleep duration, and quality | n = 160,963 participants,  n* = 2,161,067 nights of sleep tracking | Age (years), n* (%)  Adults (26 - 64), 1,298,200 (60.07) | Women, n (%)  72,862 (45.27)  Men, n (%)  88,122 (54.75) | N/A |
| Physical activity, sleep, and cardiovascular health data for 50,000 individuals from the MyHeart Counts Study [4] | iPhone | “MyHeart Counts Cardiovascular Health Study”. “Made available starting in March 2015 in the United States for iPhone 4S or newer requiring a minimum of iOS 8” | Age, Gender, Education, Clinical History Smoking Status, Heart Disease, Vascular Disease | n = 34,189 | Age (years), n (%)  <30, 6,351 (30,62)  30-39, 5,723 (27.59)  40-49, 3,696 (17.82)  50-59, 2,377 (11.46)  60-69, 1,769 (8.53)  ≥70 825 (3.97)  NA 15,998 (39.98) | Women, n (%)  4,952 (22.39)  Men, n (%)  17,151(77.55)  Other, n (%)  12 (<1)  NA, n (%)  17,901 (44.73) | Race n (%)  White  6,606 (76.15)  Black  288 (3.32)  Asian  765 (8.82)  Other  292 (<1)  Hispanic  631 (7.27) |
| Pre-symptomatic Detection of COVID-19 from Smartwatch Data [5] | Wearable devices that can detect heart rate, steps, and physiological measurements (Fitbits, Apple watches, Garmin, and others). | ” Participants with a confirmed or suspected COVID-19 infections”  Participants with high-risk exposures  Participants with “unknown respiratory illness”  Participants with no report of illnesses | Age, sex, ethnicity, height, and weight | n = 5,262  n* = 32  *” Restricted our analyses to a dataset of 32 individuals who reported a positive COVID-19 diagnosis, a diagnosis date and/or symptom onset date and (usually both; n = 28) and wearable device data appropriate for the analyses.” | Age (years), mean (SD)  44 (N/A) | n = 5,262  Women, n (%)  2,910 (55.3)  Men, n (%)  2,352 (44.7)  n* = 32  Women, n*(%)  25 (78.1)  Men, n*(%)  7 (21.9)  * denotes 32 individuals analyzed | Race n (%)  White/European 3,941(74.9)  Black  153 (2.9)  Asian  205 (3.9)  Mixed/Other/Undeclared  1010 (19.2) |
| Real-world Longitudinal Data Collected from the SleepHealth Mobile App Study [6] | iPhone with the SleepHealth Mobile app (SHMAS) | 18+ years old with iPhones  Live in the United States with fluency in English | Weight, sex, height, age, race, ethnicity, education, income, and marital status | n = 7,250 (shared broadly)  n= 2,826 (shared narrowly) | Age (years), mean (SD)  n = 7,250  36.6 (12.9)  n = 2,826  38.5 (13.1) | n = 7,250  Women, n (%)  1,508(20.8%)  Men, n (%)  5740 (79.2%)  n = 2,826  Women, n (%)  958(33.9)  Men, n (%)  1,869(66.1) | Race, n (%)  n = 7,250  White  2,541(77.9)  Asian  171(5.2)  Black/African American  93(2.9)  Multiple Race  255 (7.8)  American Indian or Alaska Native  12(0.4)  Prefer Not to Answer  36(1.1)  Other  122 (3.7)  Native Hawaiian or Other Pacific Islanders  7(0.2)  Hispanic/Latino  370(11.3)  Non-Hispanic/Non-Latino  2,846(87.2)  n = 2,826  White  609(67.4)  Asian  69(7.6)  Black/African-American  61(6.7)  Multiple Races  66(7.3)  American Indian or Alaska Native  6(0.7)  Prefer Not to Answer  37(4.1)  Other  36(40)  Native Hawaiian or Other Pacific Islander  4(0.4)  Hispanic/Latino  113(12.5)  Non-Hispanic/Non-Latino  765(84.6) |
| The Accuracy of Passive Phone Sensors in Predicting Daily Mood [7] | Android or iPhone with Wi-Fi or 3G/4G capabilities | 18+ years old with a smartphone  Able to read English  Score a five or more on the Patient Health Questionnaire-9  Indicated that the “depressive symptoms make it ‘very’ or ‘extremely’ difficult to function at work, home or socially” | Age, gender, ethnicity, race, marital status, and income  The average age of the sample was 33.4 years (SD = 10.7) and 77.8% of participants were female. The cohort was 57.5% Non‐Hispanic White, 16.2% African American/Black, and 15.1% Hispanic. A significant proportion of the participants (35.2%) reported making under $30,000 annually, and a majority (54.2%) said they couldn't make ends meet with their current income. | n =271 (BRIGHTEN sample with Android phones) | Age (years), mean (SD)  33.4(10.7) | Men, n(%)  60(22.2)  Female(%)  211(77.8) | Race,n(%)  Non-hispanic White  156(57.5)  African-American/Black  44(16.2)  Hispanic  41(15.1) |
| The Asthma Mobile Health Study, Smartphone Data Collected Using ResearchKit [8] | iPhone that can install the Asthma Health App (AHA) | 18+ years old  Have asthma  Not pregnant  Literate in English | Ethnicity, race, age, gender, income and education | n=6346 | Age(years), n(%)  18-34(60)  35-64(36)  65+(3) | Men,n(%)  1564(61)  Female,n(%)  1001(39)  NA,n(%)  3310(NA) | Race,n(%)  Black  163(5)  White  2419(69)  Other  247(7)  Multi  165(5)  Hispanic  501(14)  NA  2380(NA) |
| The mPower Study, Parkinson Disease Mobile Data Collected Using ResearchKit [9] | iPhone 4S or newer (requires a minimum of iOS 8) | 18+ years old  Individuals living in the United States  Able to read and write in English  Diagnosed with Parkinson Disease  General population (as a control)  Participants must be literate in English which poses an obstacle for non-English speaking participants | Age | n=6805 |  |  | . |
| Wearable Sensor Data and Self-reported Symptoms for COVID-19 Detection [10] | Smart devices (smartwatches and/or smartphones) capable of downloading iOS or Android research app: MyDataHelps | General population | Sex, age | n=30,529 | <35 7,052 (23.1%)  35 to 50 10,357 (33.9%)  51 to 65 9,038 (29.6%)  Over 65 3,899 (12.8%) | Women, n(%)  18,922, 62.0%  Men, n(%)  11607, 38% | Unclear as no information about racial and gender makeup was given. |
| Using a ResearchKit Smartphone App to Collect Rheumatoid  Arthritis Symptoms From Real-World Participants:Feasibility Study [11] | The PARADE app could be download from the Apple App Store in the United States. | Patients with Rheumatoid Arthritis (RA) were recruited via social media campaign in the United States. Inclusion  criteria were being 21 years of age or over, being English  speaking, living in the United States, and having a physician’s  diagnosis of RA. | Sex, age, Ethnicity, BMI, Education, Smoking History | n=399 | The mean age of included individuals was 49.2 years old with a SD of 12.48. | Women, n(%)  322 (80.7)  Men, n(%)  77 (19.3) | Ethnicity, n (%)  White 322 (80.7)  African American  16 (4.0)  Hispanic 40 (10.0)  Asian 11 (2.8)  Other 10 (2.5) |
| Harnessing wearable device data to improve state-level real-time surveillance of influenza-like illness in the USA: a population-based study [12] | Participant who used a Fitbit wearable device and who wore the same Fitbit for at least 60 days. | Targeted general population, but participants had to meet the following requirement. Participant had a self-reported birth year between 1930 and 2004, height greater than 1 m, and weight greater than 20 kg. The study chose to focus on participants in the following five states: California, Texas, New York, Illinois, and Pennsylvania. | Sex, Age, BMI | n=47, 249 (after screening process) | The mean age of included individuals was 42.7 years old. | Women, n(%)  28465 (60.2%)  Men, n(%)  18594 (39.4%)  Unknown, n(%)  190 (0·4%) | Unclear as no information about racial and gender makeup was given |
| Using Mobile Apps to Assess and Treat Depression in Hispanic and Latino Populations: Fully Remote Randomized Clinical Trial [13] | Smartphone or tablet | Targeted Hispanic/Latino adults with mild to moderate depression through social media, Hispanic/Latino Catholic ministries, and posting on Craiglist and provided monetary compensation | Age, Income, Education, Race, Income satisfaction, Marital status | n = 345 | 18-30 137(40.2)  31-40 101(29.6)  41-50 74(21.7)  51-60 23(6.7)  61-70 5(1.5)  >70 1(0.3) | Women, n(%)  266 (77.1%)  Men, n(%)  79 (22.9) | Hispanic/Latinos 106 (30.7)  Non-Hispanic white 184 (53.3)  African-Americna/black 25 (7.2)  American Indian/Alaskan Native 3 (0.9)  Asian 24 (7.0)  Other 3 (0.9) |
| Learning endometriosis phenotypes from patient-generated data [14] | Phendo app available for both iOS and Android based phones | Recruitment of women through patient advocacy groups and active recruitment efforts such as social media, celebrity endorsement, emails, radio, etc. | Age, Gender, BMI, Race/ethnicity, Education, Living environment | n = 4,368 | 30.29 (7.0) | Male, 3 (0.1%)  Other, 40 (0.9%)  Female, 4308 (99.0%) | Native American, 29 (0.7%)  Black, non-Hispanic, 101 (2.3%)  Asian, 111 (2.6%)  Hispanic, 215 (4.9%)  Other, 290 (6.7%)  White, non-Hispanic, 3604 (82.9%) |
| Evaluating the Utility of Smartphone-Based Sensor Assessments in Persons With Multiple Sclerosis in the Real-World Using an App (elevateMS): Observational, Prospective Pilot Digital Health Study [15] | Smartphone capable of downloading elevateMS app | Patient-centered design with individuals with multiple sclerosis (self-referred or clinic-referred) or without multiple sclerosis | age, gender, race, education, health insurance, employment status, geographic location | n = 629 | mean (SD) age of 39.34 (11.41), 45.20 (11.64), and 48.93 (11.20) years in the control, self-referred, and clinic-referred cohorts, respectively | (self-referred MS excluding missingness)  Women, n(%)  154 (73.3%)  Men, n(%)  56 (26.7.4%) | Ethnicity, n (%)  White 182 (85.4)  African American  13 (6.1)  Hispanic 9 (4.2)  Asian 4 (1.9)  Other 5 (2.3)  s |

[1] N. L. Spartano *et al.*, “Comparison of On-Site Versus Remote Mobile Device Support in the Framingham Heart Study Using the Health eHeart Study for Digital Follow-up: Randomized Pilot Study Set Within an Observational Study Design,” *JMIR Mhealth Uhealth*, vol. 7, no. 9, Sep. 2019, doi: 10.2196/13238.

[2] B. L. Smarr *et al.*, “Feasibility of continuous fever monitoring using wearable devices,” *Scientific Reports*, vol. 10, no. 1, Art. no. 1, Dec. 2020, doi: 10.1038/s41598-020-78355-6.

[3] R. Robbins, M. Affouf, A. Seixas, L. Beaugris, G. Avirappattu, and G. Jean-Louis, “Four-Year Trends in Sleep Duration and Quality: A Longitudinal Study Using Data from a Commercially Available Sleep Tracker,” *J Med Internet Res*, vol. 22, no. 2, Feb. 2020, doi: 10.2196/14735.

[4] S. G. Hershman *et al.*, “Physical activity, sleep and cardiovascular health data for 50,000 individuals from the MyHeart Counts Study,” *Sci Data*, vol. 6, Apr. 2019, doi: 10.1038/s41597-019-0016-7.

[5] T. Mishra *et al.*, “Pre-symptomatic detection of COVID-19 from smartwatch data,” *Nature Biomedical Engineering*, pp. 1–13, Nov. 2020, doi: 10.1038/s41551-020-00640-6.

[6] S. Deering *et al.*, “Real-world longitudinal data collected from the SleepHealth mobile app study,” *Sci Data*, vol. 7, Nov. 2020, doi: 10.1038/s41597-020-00753-2.

[7] A. Pratap *et al.*, “The accuracy of passive phone sensors in predicting daily mood,” *Depression and Anxiety*, vol. 36, no. 1, pp. 72–81, 2019, doi: https://doi.org/10.1002/da.22822.

[8] Y.-F. Y. Chan *et al.*, “The asthma mobile health study, smartphone data collected using ResearchKit,” *Sci Data*, vol. 5, May 2018, doi: 10.1038/sdata.2018.96.

[9] B. M. Bot *et al.*, “The mPower study, Parkinson disease mobile data collected using ResearchKit,” *Scientific Data*, vol. 3, no. 1, Art. no. 1, Mar. 2016, doi: 10.1038/sdata.2016.11.

[10] G. Quer *et al.*, “Wearable sensor data and self-reported symptoms for COVID-19 detection,” *Nature Medicine*, pp. 1–5, Oct. 2020, doi: 10.1038/s41591-020-1123-x.

[11] M. Crouthamel *et al.*, “Using a ResearchKit Smartphone App to Collect Rheumatoid Arthritis Symptoms From Real-World Participants: Feasibility Study,” *JMIR Mhealth Uhealth*, vol. 6, no. 9, Sep. 2018, doi: 10.2196/mhealth.9656.

[12] J. M. Radin, N. E. Wineinger, E. J. Topol, and S. R. Steinhubl, “Harnessing wearable device data to improve state-level real-time surveillance of influenza-like illness in the USA: a population-based study,” *The Lancet Digital Health*, vol. 2, no. 2, pp. e85–e93, Feb. 2020, doi: 10.1016/S2589-7500(19)30222-5.

[13] A. Pratap *et al.*, “Using Mobile Apps to Assess and Treat Depression in Hispanic and Latino Populations: Fully Remote Randomized Clinical Trial,” *J Med Internet Res*, vol. 20, no. 8, p. e10130, Aug. 2018, doi: 10.2196/10130.

[14] I. Urteaga, M. McKillop, and N. Elhadad, “Learning endometriosis phenotypes from patient-generated data,” *npj Digit. Med.*, vol. 3, no. 1, pp. 1–14, Jun. 2020, doi: 10.1038/s41746-020-0292-9.

[15] A. Pratap *et al.*, “Evaluating the Utility of Smartphone-Based Sensor Assessments in Persons With Multiple Sclerosis in the Real-World Using an App (elevateMS): Observational, Prospective Pilot Digital Health Study,” *JMIR mHealth and uHealth*, vol. 8, no. 10, p. e22108, Oct. 2020, doi: 10.2196/22108.
