# Supplementary material for: Demographic Imbalances Resulting From the Bring-Your-Own-Device Study Design
Source: JMIR Mhealth Uhealth. 2022 Apr 8;10(4):e29510. doi: 10.2196/29510 (PMC9034431; doi:10.2196/29510)
Supplement: Multimedia Appendix 2 [file mhealth_v10i4e29510_app2.docx]

**Multimedia Appendix 2.** Sex and Race/Ethnicity Reported by Various BYOD Studies, Census, and National Vital Statistics System.

|  | | | Sex | | | | Reported Race and Ethnicity | | | |  | |
| --- | --- | --- | --- | --- | --- | --- | --- | --- | --- | --- | --- | --- |
| Study Name | Women | Men | White | Black | Asian | Hispanic/  Latino | | Non-Latino | Other | P-value of Chi-Squared Test of Race and Ethnicity between US Demographics and Study Population | |  |
| Framingham Heart Study [1] | 59 | 41 | N/A | N/A | N/A | N/A | | N/A | N/A | N/A | |  |
| TemPredict* [2] | 34 | 66 | 81 | 0 | 4 | 17 | | 83 | 15 | P < .001 | |  |
| Sleep Cycle [3] | 45.27 | 54.75 | N/A | N/A | N/A | N/A | | N/A | N/A | N/A | |  |
| MyHeartCounts [4] | 22.39 | 77.55 | 76.15 | 3.32 | 8.82 | 7.27 | | 19.87 | 4.44 | P < .001 | |  |
| MyPHD [5] | 55.3 | 44.7 | 74.9 | 2.9 | 3.9 | 0 | | 0 | 18.3 | P < .001 | |  |
| SleeHealth Mobile App* [6] | 20.8 | 79.2 | 77.9 | 2.9 | 5.2 | 11.3 | | 87.2 | 3.7 | P < .001 | |  |
| Predicting Daily Mood [7] | 77.8 | 22.2 | 57.5 | 16.2 | N/A | 15.1 | | N/A | N/A | N/A | |  |
| Asthma Health App [8] | 39 | 61 | 69 | 5 | N/A | 14 | | N/A | 7 | N/A | |  |
| MyDataHelps (COVID-19 Study) [9] | 62 | 38 | N/A | N/A | N/A | N/A | | N/A | N/A | N/A | |  |
| PARADE App [10] | 80.7 | 19.3 | 80.7 | 4 | 2.8 | 10 | | N/A | N/A | P < .001 | |  |
| Multiple Sclerosis in the Real-World Using an App (elevateMS) [11] | 73.3 | 26.7 | 85.4 | 6.1 | 1.9 | 4.2 | | 95.7 | 2.3 | P < .001 | |  |
| Phendo Endometris Research App [12] | 99 | 3 | 82.9 | 2.3 | 2.6 | 4.9 | | 95.1 | 6.7 | P < .001 | |  |
| Brighten [13] | 77.1 | 22.9 | 53.3 | 7.2 | 7.0 | 30.7 | | 69.3 | 0.9 | P = 0.008 | |  |
| Influenza-like Illness Study [14] | 60.2 | 39.4 | N/A | N/A | N/A | N/A | | N/A | N/A | N/A | |  |
| All of Us (Fitbit) [15] | 70 | 28 | 82.4 | 4.53 | 3.05 | 6.38 | | N/A | 2.42 | P < .001 | |  |
| All of Us (All Participants) [15] | 60.1 | 37.7 | 51.5 | 21.24 | 3.26 | 18.8 | | N/A | N/A | P = 0.072 | |  |
| CovIdentify | 67.75 | 32.2 | 87.55 | 3.61 | 3.28 | 4 | | N/A | N/A | P < .001 | |  |
| U.S. Census Demographics [16] | 50.8 | 49.2 | 60.1 | 13.4 | 5.9 | 18.5 | | 81.5 | N/A | P = 1.0 | |  |
| COVID-19 Positive Cases [16] | N/A | N/A | 34.79 | 21.81 | 3.85 | 33.38 | | N/A | N/A | P < .001 | |  |
| Deaths in the U.S. due to COVID-19 [17] | N/A | N/A | 53.56 | 23.26 | 4.95 | 17.08 | | N/A | N/A | P = 0.042 | |  |
|  |  |  |  |  |  |  | |  |  |  | |  |

[1] N. L. Spartano *et al.*, “Comparison of On-Site Versus Remote Mobile Device Support in the Framingham Heart Study Using the Health eHeart Study for Digital Follow-up: Randomized Pilot Study Set Within an Observational Study Design,” *JMIR Mhealth Uhealth*, vol. 7, no. 9, Sep. 2019, doi: 10.2196/13238.

[2] B. L. Smarr *et al.*, “Feasibility of continuous fever monitoring using wearable devices,” *Scientific Reports*, vol. 10, no. 1, Art. no. 1, Dec. 2020, doi: 10.1038/s41598-020-78355-6.

[3] R. Robbins, M. Affouf, A. Seixas, L. Beaugris, G. Avirappattu, and G. Jean-Louis, “Four-Year Trends in Sleep Duration and Quality: A Longitudinal Study Using Data from a Commercially Available Sleep Tracker,” *J Med Internet Res*, vol. 22, no. 2, Feb. 2020, doi: 10.2196/14735.

[4] S. G. Hershman *et al.*, “Physical activity, sleep and cardiovascular health data for 50,000 individuals from the MyHeart Counts Study,” *Sci Data*, vol. 6, Apr. 2019, doi: 10.1038/s41597-019-0016-7.

[5] T. Mishra *et al.*, “Pre-symptomatic detection of COVID-19 from smartwatch data,” *Nature Biomedical Engineering*, pp. 1–13, Nov. 2020, doi: 10.1038/s41551-020-00640-6.

[6] S. Deering *et al.*, “Real-world longitudinal data collected from the SleepHealth mobile app study,” *Sci Data*, vol. 7, Nov. 2020, doi: 10.1038/s41597-020-00753-2.

[7] A. Pratap *et al.*, “The accuracy of passive phone sensors in predicting daily mood,” *Depression and Anxiety*, vol. 36, no. 1, pp. 72–81, 2019, doi: https://doi.org/10.1002/da.22822.

[8] Y.-F. Y. Chan *et al.*, “The asthma mobile health study, smartphone data collected using ResearchKit,” *Sci Data*, vol. 5, May 2018, doi: 10.1038/sdata.2018.96.

[9] B. M. Bot *et al.*, “The mPower study, Parkinson disease mobile data collected using ResearchKit,” *Scientific Data*, vol. 3, no. 1, Art. no. 1, Mar. 2016, doi: 10.1038/sdata.2016.11.

[10] G. Quer *et al.*, “Wearable sensor data and self-reported symptoms for COVID-19 detection,” *Nature Medicine*, pp. 1–5, Oct. 2020, doi: 10.1038/s41591-020-1123-x.

[11] A. Pratap *et al.*, “Evaluating the Utility of Smartphone-Based Sensor Assessments in Persons With Multiple Sclerosis in the Real-World Using an App (elevateMS): Observational, Prospective Pilot Digital Health Study,” *JMIR mHealth and uHealth*, vol. 8, no. 10, p. e22108, Oct. 2020, doi: 10.2196/22108.

[12] I. Urteaga, M. McKillop, and N. Elhadad, “Learning endometriosis phenotypes from patient-generated data,” *npj Digit. Med.*, vol. 3, no. 1, pp. 1–14, Jun. 2020, doi: 10.1038/s41746-020-0292-9.

[13] A. Pratap *et al.*, “Using Mobile Apps to Assess and Treat Depression in Hispanic and Latino Populations: Fully Remote Randomized Clinical Trial,” *J Med Internet Res*, vol. 20, no. 8, p. e10130, Aug. 2018, doi: 10.2196/10130.

[14] J. M. Radin, N. E. Wineinger, E. J. Topol, and S. R. Steinhubl, “Harnessing wearable device data to improve state-level real-time surveillance of influenza-like illness in the USA: a population-based study,” *The Lancet Digital Health*, vol. 2, no. 2, pp. e85–e93, Feb. 2020, doi: 10.1016/S2589-7500(19)30222-5.

[15] “All of Us Research Program Expands Data Collection Efforts with Fitbit,” *National Institutes of Health (NIH) — All of Us*, Aug. 20, 2020. https://allofus.nih.gov/news-events-and-media/announcements/all-us-research-program-expands-data-collection-efforts-fitbit (accessed Mar. 23, 2021).

[16] “U.S. Census Bureau QuickFacts: United States.” https://www.census.gov/quickfacts/fact/table/US/RHI125219 (accessed Jul. 15, 2020).

[17] “Provisional Death Counts for Coronavirus Disease 2019 (COVID-19),” Dec. 08, 2021. https://www.cdc.gov/nchs/nvss/vsrr/covid19/index.htm (accessed Dec. 08, 2021).
