# Supplementary material for: Demographic Imbalances Resulting From the Bring-Your-Own-Device Study Design
Source: JMIR Mhealth Uhealth. 2022 Apr 8;10(4):e29510. doi: 10.2196/29510 (PMC9034431; doi:10.2196/29510)
Supplement: Multimedia Appendix 3 [file mhealth_v10i4e29510_app3.docx]

**Multimedia Appendix 3.** Wearable Device Ownership by Race or Ethnicity for CovIdentify Case Study

|  | Race and Ethnicity Count by Wearable Device | | | |
| --- | --- | --- | --- | --- |
| Watch | White | Black | Asian | Hispanic |
| Fitbit | 1257 | 40 | 49 | 52 |
| Apple | 1179 | 63 | 68 | 54 |
| Garmin | 1847 | 34 | 77 | 123 |
| Samsung | 107 | 24 | 7 | 14 |
| Polar | 22 | 0 | 0 | 2 |
| Suunto | 10 | 0 | 1 | 1 |
| Withings | 19 | 0 | 3 | 2 |
| Withit | 2 | 0 | 0 | 0 |
| Other | 174 | 11 | 15 | 17 |
