# Supplementary material for: Demographic Imbalances Resulting From the Bring-Your-Own-Device Study Design
Source: JMIR Mhealth Uhealth. 2022 Apr 8;10(4):e29510. doi: 10.2196/29510 (PMC9034431; doi:10.2196/29510)
Supplement: Multimedia Appendix 4 [file mhealth_v10i4e29510_app4.docx]

**Multimedia Appendix 4.** Wearable Device Ownership of iPhone (iOS) Users by Race or Ethnicity for CovIdentify Case Study

|  | Race and Ethnicity Count by Wearable Device for iOS Users | | | |
| --- | --- | --- | --- | --- |
| Watch | White | Black | Asian | Hispanic |
| Fitbit | 29 | 2 | 2 | 0 |
| Apple | 75 | 6 | 4 | 0 |
| Garmin | 28 | 0 | 0 | 0 |
| Samsung | 0 | 1 | 0 | 0 |
| Polar | 0 | 0 | 0 | 0 |
| Suunto | 0 | 0 | 0 | 0 |
| Withings | 3 | 0 | 3 | 0 |
| Withit | 0 | 0 | 0 | 0 |
| Other | 32 | 4 | 2 | 0 |
